# A187 RISK FACTORS OF CLINICAL RELAPSES IN PEDIATRIC LUMINAL CROHN’S DISEASE, A RETROSPECTIVE COHORT STUDY

**DOI:** 10.1093/jcag/gwab049.186

**Published:** 2022-02-21

**Authors:** S Sassine, L Djani, C Cambron-Asselin, M Savoie-Robichaud, Y Lin, S Fadela Zekhnine, M Qaddouri, K Grzywacz, V Groleau, M Dirks, É Drouin, U Halac, V Marchand, C Girard, O Courbette, N Patey, D Dal Soglio, C Deslandres, P Jantchou

**Affiliations:** Centre Hospitalier Universitaire Sainte-Justine Centre de Recherche, Montreal, QC, Canada

## Abstract

**Background:**

Few risk factors are associated with the risk of relapses of Crohn’s disease in children.

**Aims:**

The aims of this retrospective cohort study were to describe the rate of relapses in children with Crohn’s disease, its evolution over the past decade and to determine risk factors associated with relapse.

**Methods:**

Patients under 18 years old and diagnosed between 2009 and 2019 were included. Patients clinical, endoscopic, histological, and laboratory characteristics, as well as their treatments, where collected from their medical records and the prospective CHU Sainte-Justine inflammatory bowel disease registry. Survival analyses and Cox regression models were used to assess the impact of those risk factors on relapse.

**Results:**

639 patients were included. There was a decrease in the clinical relapse rate over the past decade: 70.9% of patients diagnosed between 2009 and 2014 experienced a relapse compared to 49.1% of patients diagnosed between 2015 and 2019 (p<0.0001). The following variables were associated with clinical relapse: female sex (adjusted hazard ratio (aHR)= 1.51, p=0.0009), high PCDAI (aHR= 1.02, p=0.04) and SES-CD (aHR= 1.03, p=0.03) scores at diagnosis, upper digestive tract involvement (aHR= 1.59, p=0.0003), exposure to oral 5-ASA (aHR= 1.91, p=0.0003), use of immunomodulatory agents compared to TNF-alpha inhibitors (methotrexate aHR= 1.91, p=0.0006; thiopurines aHR= 2.06, p<0.0001), presence of granulomas (aHR= 1.27, p=0.04) and increased eosinophils on intestinal biopsies (aHR= 1.34, p=0.02), high levels of C-reactive protein (aHR= 1.01, p<0.0001) and fecal calprotectin (aHR=1.09, p<0.0001) during clinical remission and low serum infliximab levels during maintenance (aHR for mean serum infliximab level under 7ug/mL = 2.48, p=0.005).

**Conclusions:**

Relapse risk was significantly associated with baseline clinical, endoscopic, histological and laboratory data and treatment strategies. These results could help better select treatment options for pediatric Crohn’s disease at induction and maintenance.

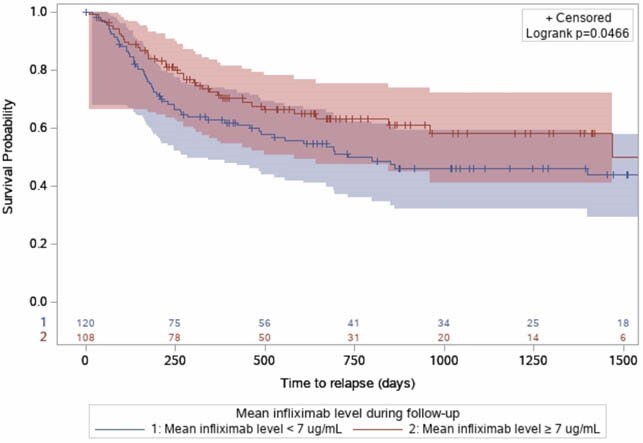

Kaplan-Meier curve representing patients time to relapse according to the mean infliximab level in post-induction.

**Funding Agencies:**

NoneFonds Recherche Santé Québec / Fondation du CHU Sainte-Justine

